# Testing hypotheses of skull function with comparative finite element analysis: three methods reveal contrasting results

**DOI:** 10.1242/jeb.249747

**Published:** 2025-02-25

**Authors:** D. Rex Mitchell, Stephen Wroe, Meg Martin, Vera Weisbecker

**Affiliations:** ^1^ College of Science and Engineering, Flinders University, GPO Box 2100, Adelaide, SA 5001, Australia; ^2^ Australian Research Council Centre of Excellence for Australian Biodiversity and Heritage, Wollongong, NSW 2522, Australia; ^3^ School of Environmental and Rural Science, University of New England, Armidale, NSW 2351, Australia; ^4^ Collections and Research, Western Australian Museum, Locked Bag 49, Welshpool, WA 6986, Australia

**Keywords:** Bite force, Feeding biomechanics, Finite element analysis, Mechanical advantage, Potoroidae, Scaling

## Abstract

Comparative finite element analysis often involves standardising aspects of models to test equivalent loading scenarios across species. However, regarding feeding biomechanics of the vertebrate skull, what is considered ‘equivalent’ can depend on the hypothesis. Using 13 diversely shaped skulls of marsupial bettongs and potoroos (Potoroidae), we demonstrate that scaling muscle forces to standardise specific aspects of biting mechanics can produce clearly opposing comparisons of stress or strain that are differentially suited to address specific kinds of hypotheses. We therefore propose three categories of hypotheses for skull biting mechanics, each involving a unique method of muscle scaling to produce meaningful results: those comparing (1) the skull's efficiency in distributing muscle forces to the biting teeth, via standardising input muscle force to skull size, (2) structural biting adaptation through standardising mechanical advantage to simulate size-adjusted, equivalent bites and (3) feeding ecology affected by size, such as niche partitioning, via standardising bite reaction force.

## Teaser

**Summary:** Common approaches for scaling muscle forces in skull finite element models might not always offer reliable results for all hypotheses: a framework for selecting the appropriate method is provided.

## INTRODUCTION

Comparisons of mechanical performance provide valuable insights into how anatomical structures have evolved in response to functional demands. These comparisons not only reveal how species have specialised to fulfil their ecological roles but also shed light on evolutionary relationships and biomechanical trade-offs (Pérez-Ramos et al., 2020; Rayfield, 2007; Sansalone et al., 2024). As often the first point of contact between an animal and its sustenance, mechanical performance of the feeding system in particular is an important determinant of an animal's relative fitness and survival (Ross and Iriarte-Diaz, 2014).

Over the last few decades, computer-assisted biomechanical methods and their application to the study of skeletal function have provided researchers with new tools to test hypotheses related to dietary adaptation. In particular, finite element analysis (FEA), a computational engineering technique used to simulate the behaviour of geometrically complex structures under load, has become established as a powerful tool in modelling the relationship between shape and function in the vertebrate skeleton (e.g. Bright, 2014; Korioth and Versluis, 1997; Rayfield, 2007; Wroe, 2010).

Complex biological structures in reality have infinite points of reference and are therefore computationally infeasible. FEA addresses this problem by simplifying complex structures into a large but definable number of simple geometric elements. These elements form a contiguous mesh and are joined at their corners by ‘nodes’ with specific coordinates in digital space. The elements can be assigned a range of material properties, and simulations of loading involve the application of forces and constraints at specific nodes located at regions of anatomical and functional significance. With respect to the skull and assessing its ability to bite, jaw adductor muscle forces are simulated, while the temporomandibular joints and bite point(s) are constrained. These can then yield joint reaction forces and bite reaction forces from solved models. Other metrics returned by the models include the mechanical advantage, or leverage, of bite force production (bite reaction force divided by input muscle force), strain (i.e. deformation, or bending of the structure, defined as change in length divided by initial length), and stress (force per unit of area). These are key variables in determining mechanical performance and are useful for quantifying morphological adaptation because the methods used to obtain them are non-invasive and repeatable (Wroe, 2010).

In most clinical contexts, researchers ideally aim to ensure that the stress and strain values obtained from finite element models (FEMs) represent real-world scenarios as accurately as possible. This is achieved through muscle dissections and *in vivo* validations of strain magnitudes obtained from organic samples for comparison with values obtained from solved FEMs. But muscle data and validations are difficult to obtain for many species, and impossible for extinct species. To allow for this uncertainty, a simplified comparative approach can be used for non-clinical research whereby the material properties applied to elements are often homogeneous and the simulated forces are arbitrarily defined but then standardised for all models to have consistent ratios of force to size (Dumont et al., 2009, 2011a). This procedure is not intended to produce mechanical data that reflect biological reality, but instead aims to provide a relative comparison of different species performing the same simulated action. In the context of comparing feeding biomechanics through overall performance of the skull, many studies have shown that allocating homogeneous, isotropic material properties results in negligible differences in broad patterns of stress and strain compared with multi-property models (e.g. Fitton et al., 2015; Panagiotopoulou et al., 2011; Strait et al., 2010; Walmsley et al., 2013). Through these simplified model constructions, lower stress or strain magnitudes in one skull compared with another skull would typically be interpreted as representative of better performance through greater resistance to bending of the bone and potential injury (e.g. Dumont et al., 2011a; Ledogar et al., 2022; Mitchell et al., 2018; Wroe, 2010). Comparative FEA has been used in this way to great effect across diverse extant and extinct vertebrates, particularly to assess feeding biomechanics of the vertebrate skull (Attard et al., 2011; Cook et al., 2021; Cox et al., 2011, 2015; Dumont et al., 2011a,b; Ferrara et al., 2011; Figueirido et al., 2014; Ledogar et al., 2016, 2018, 2022; Mitchell, 2019; Mitchell et al., 2018; Mitchell and Wroe, 2019; Oldfield et al., 2011; Rayfield, 2011; Slater et al., 2009; Smith et al., 2015b; Strait et al., 2009; Tseng, 2008; Tseng et al., 2011; van Heteren et al., 2021; Wroe, 2007; Wroe et al., 2007, 2013). Through these studies, we have gained valuable insights into the relationship between skull morphology and mechanical performance, with applications towards animal behaviour, conservation, ecology, evolution and palaeontology.

The goal of the comparative approach is to standardise parameters as much as possible to subject each model to equivalent, or near equivalent, loading conditions. However, what constitutes equivalency might differ depending on the hypothesis; and various standardising regimes used by researchers might not be adequate for answering all possible questions focused on how well different species perform during biting. For example, research using standardised muscle force on larger samples of models has identified inconsistencies resulting from interspecific variation in mechanical advantage that can impact strain magnitudes (Mitchell et al., 2018). Furthermore, the roles that size and shape both play in overall function of the skull (Chamoli and Wroe, 2011; Mitchell et al., 2024c) suggest that common approaches used to remove variation in size are potentially not appropriate for all questions relating to animal ecology and feeding behaviour.

Because most hypotheses of skull biomechanics focus on the relationship between shape and performance (i.e. functional morphology), without consideration of how size impacts on cranial performance, it has been recommended that variation due to size be removed through standardising the ratios of size and muscle force (Dumont et al., 2009, 2011a; Strait et al., 2009). There are two common methods that aim to achieve this. The first is to scale all models to the same size (usually by standardising surface areas) and apply identical muscle forces to all models (Attard et al., 2011; Dumont et al., 2009; Wroe et al., 2010). The second is to scale the muscle forces (McHenry et al., 2007). This is most often done using skull volume to a two-thirds power (Cook et al., 2021; Ledogar et al., 2016, 2018; Mitchell, 2019; Smith et al., 2015a,b; Strait et al., 2010). This second approach normalises forces following the established relationship between muscle force and size (Alexander, 1985). Either approach of standardising ratios of skull size and muscle forces is intended to provide a means of comparing the ability of skull shapes to convert muscle force to bite force. However, standardising input muscle force is just the first of three standardisations that can be enacted on the jaw lever system in FEA. The other two are standardising mechanical advantage and standardising bite reaction force. Standardising each of these might produce different comparisons of stress or strain, especially if the sample has appreciable variation in mechanical advantage or size.

In studies of feeding biomechanics, mechanical advantage refers to how much muscle force can be converted to bite force. In its simplest terms, this is defined by the relative lengths of the in-lever (distance of the average muscle force vector from the temporomandibular joint) and out-lever (distance of the biting tooth from the temporomandibular joint). All else being equal, skulls with orthognathic proportions, or ‘shorter faces’, therefore tend to transfer more input muscle force to the biting teeth, representing greater mechanical advantage (Goswami et al., 2010; Mitchell et al., 2024c). Selection for mechanical advantage is considered one of many important determinants of mammalian skull diversity (Dumont et al., 2009, 2014; Ledogar et al., 2022; Mitchell et al., 2024c), with jaw length playing a key role in how efficient a jaw lever system is.

Standardising input muscle forces may be reasonable for calculating mechanical advantage and assessing stress or strain of the skull under equivalent muscle loading, but this method might not account for variation in mechanical advantage. Consequently, models of species with lower mechanical advantage might produce weaker bites from the same input muscle force and therefore experience lower stress and deformation throughout the facial skeleton. Because lower strain magnitudes are often interpreted to represent morphology better suited to resist deformation and injury during biting, there is a risk that longer faces with lower strains could be misinterpreted as indicating that the species are better adapted to forceful biting than they are. Conversely, skulls with high mechanical advantage would produce stronger bites and therefore experience greater deformation of the facial skeleton than should be expressed during equivalent biting behaviours (Mitchell et al., 2018). Studies that examine how efficiently different skull shapes support equivalent bites, relative to size, might therefore benefit from standardising mechanical advantage.

**Fig. 1.**
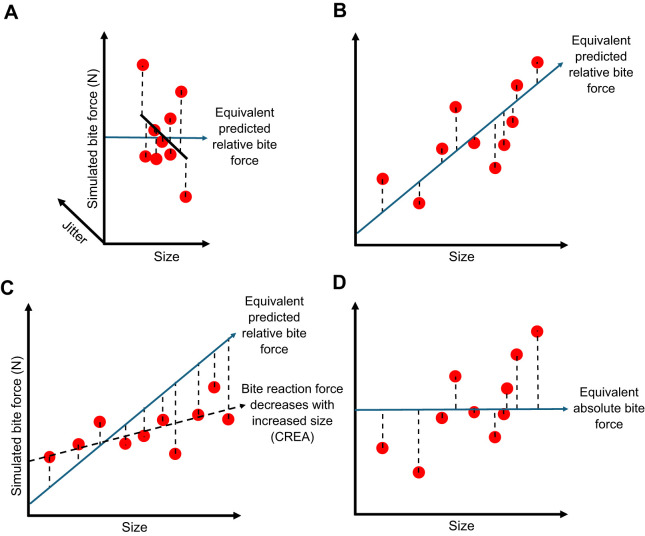
**Methods of muscle force scaling in comparative finite element analysis (FEA).** (A) When models initially scaled to the same size have the same input muscle forces applied, all bite reaction forces plot on a single point of the size axis but show variation due to mechanical advantage (jitter variation on a *z*-axis added for clarity). Muscle forces can be rescaled such that each model produces the same bite reaction force. As models have been scaled to the same size, this approach aims to simulate relative (size-independent) bite force. (B) When models retain their natural size and instead have muscle forces scaled to size, bite reaction forces from initial simulations are plotted against size. The mean mechanical advantage of the sample is multiplied by initial muscle forces to predict equivalent relative bite force. Initial muscle forces can then be rescaled to produce the same relative bite force. (C) When samples exhibit craniofacial evolutionary allometry (CREA), a pattern of decreasing mechanical advantage with size will often be seen. For such samples, standardising mechanical advantage requires greater input muscle force applied to larger species and reduced input muscle force applied to smaller species. (D) To factor size into function for hypotheses relating to ecology, muscle forces on models with original size retained can also be rescaled to produce the same absolute bite force. In this case, we use the mean bite force from the sample, but the chosen bite force is arbitrary.

There are two main methods that aim to standardise mechanical advantage. The first is done on models that have been scaled to equivalent size, by rescaling the initial muscle forces to result in a common bite force (Fig. 1A) (Attard et al., 2011; Oldfield et al., 2011; Wroe et al., 2010). The goal is to ensure that all models are performing an equivalent action, for their size, and the stress and strain magnitudes therefore adequately reflect an equivalent action of the jaw, when adjusted for size. The second method can be applied to models of their natural size that have had their initial muscle forces scaled to volume to the two-thirds power. This involves rescaling initial input muscle forces to produce bite reaction forces expected from a standardised mechanical advantage (Fig. 1B) (Mitchell et al., 2018; Mitchell and Wroe, 2019). For the many taxa that exhibit an increase in face length with increased size [i.e. a craniofacial evolutionary allometry (CREA) pattern] (Cardini et al., 2015; Cardini and Polly, 2013; Mitchell et al., 2024b,c; Tamagnini et al., 2017), mechanical advantage will often decrease with increasing size. The predictive regression line of standardised mechanical advantage would therefore follow a path of increasing bite reaction force when compared with the initial loadings, indicating that larger individuals would need greater muscle forces applied to achieve equivalent relative biting actions (Fig. 1C). Both of the above approaches aim to account for variation in mechanical advantage and might therefore more accurately compare the performance of the skull for hypotheses relating to skull shape and biting adaptation. However, these approaches are not necessarily adequate for hypotheses of a more ecological focus, such as how well animals of different sizes can bite the same piece of food. Here, we propose that such hypotheses also might benefit from a third standardising technique that includes size variation in the analysis. This requires the standardisation of absolute bite reaction forces.

Dumont et al. (2009) advised the disentangling of size and shape for comparative finite element modelling. Their perspective came from a foundational prioritisation of shape as the predominant indicator of function and emphasised the importance of shape differences in relation to mechanical performance. However, size is a crucial determinant of an animal's skull structure and absolute function in an ecosystem (Alexander, 1985; Chamoli and Wroe, 2011; Doube et al., 2011; Zelditch and Swiderski, 2023; Mitchell et al., 2024c). Adaptive trade-offs in the mammalian cranium across size ranges might suggest that size and morphology should not be separated when considering adaptation to environmental parameters that can oftentimes remain constant regardless of size (Marcy et al., 2024; Mitchell et al., 2024b,c). In particular, all else being equal, if larger species can bite harder than smaller species owing to their larger skulls, jaw muscles and teeth, the skull of a larger species will more easily bite into an object of a given size and composition than the skull of a smaller species. This potentially allows the evolution of lower mechanical advantage in larger species (e.g. through elongation of the facial proportions) without sacrificing absolute bite force (Zelditch et al., 2017; Mitchell et al., 2024c) and means that the degree of biting adaptation seen in a skull is likely not independent of the size of the animal.

Because both size and shape jointly determine the mechanical capabilities of an animal under natural settings, we suggest that size should be considered in hypotheses relating to comparisons of behavioural ecology, competition, niche differentiation and overall potential interactions and impacts on the environment. This would involve scaling muscle forces on models of their natural size to produce the same absolute bite reaction force (Fig. 1D). Surprisingly, this approach has almost never been taken (but see Fitton et al., 2015). But by factoring size into the muscle scaling protocol of skull FEA, thus representing absolute (size-dependent) bite force comparisons, we would expect to see stress and strain magnitudes across models that can compare quite differently to those offered by relative (size-independent) bite force simulations. For example, smaller skull models should exhibit greater deformation, while larger models should exhibit reduced deformation compared with size-adjusted simulations on the same models.

Here, we show that all three standardising approaches produce different estimations of cranial stress or strain (stress and strain are directly proportional when using homogeneous, isotropic models). We chose the family of Potoroidae, including bettongs and potoroos, as an ideal group to compare results from the three methods. Potoroids exhibit a suitably large range of elongation of the facial skeleton that appears to be independent of size, suggesting strong selection on mechanical advantage (Mitchell et al., 2018). All extant species specialise on highly digestible foods with discrete and constant mechanical properties, such as fungal sporocarps (truffles), roots, tubers, seeds, nuts, fruits and insects, and these food groups comprise the diets of each species to varying proportions (Mitchell et al., 2024a; Seebeck et al., 1989). Importantly, species with a greater focus on softer fungi tend to have longer snouts, while shorter faces tend to be accompanied by diets with harder foods, such as nuts, seeds and roots (Mitchell et al., 2024a, 2018). To demonstrate the predictive power of our approach, we also included an extinct species, *Caloprymnus campestris*, which had a small skull for a potoroid, but particularly robust craniofacial proportions.

Specifically, we aimed to test three hypotheses: (1) when applying only the initial standardised input muscle forces, variation in mechanical advantage can confound strain magnitudes for assessments of equivalent biting ability, such that species with long faces will have lower strain and short-faced species will have higher strain – this would be contrary to what should be expected from their diets; (2) when muscle forces are rescaled to standardise mechanical advantage, and therefore produce equivalent bite forces relative to size, skulls with shorter snouts (i.e. greater mechanical advantage) will exhibit lower strain than skulls with longer snouts; and (3) when rescaling muscle forces to standardise absolute bite reaction forces, the model of the smaller, more robust cranium of *C. campestris* will experience higher mean strain than other species with similarly efficient skulls, suggesting that it was not as capable of biting the same foods as larger species with similarly robust skull anatomy.

## MATERIALS AND METHODS

Our sample included 13 intact, adult skull specimens from the South Australian Museum and the Queensland Museum: two *Aepyprymnus rufescens* (J. E. Gray 1837) (SAM M2750 and SAM M12026), two *Bettongia gaimardi* (Desmarest 1822) (SAM M7384 and SAM M7389), two *Bettongia lesueur* (Quoy & Gaimard 1824) (SAM M1705 and SAM M18492), two *Bettongia penicillata* J. E. Gray 1837 (SAM M8285 and SAM M11252), two *Bettongia tropica* Wakefield 1967 (QM JM10030 and QM JM12495), two *Potorous tridactylus* (Kerr 1792) (SAM M7381 and SAM M9013), and one *Caloprymnus campestris* (Gould 1843) (SAM M3257). We micro-computed tomography (μCT) scanned all specimens using the Flinders Tonsley Nikon XT H 225ST CT Scanner (Large-Volume Micro-CT System), with isotropic pixel sizes ranging from 20 to 25 µm.

We generated 3D surface meshes of the crania and mandibles from the μCT data in Mimics (Materialise v.26.0). For each model, the cranium was centred and then oriented to align the temporomandibular joints and dental arcades with the *y*-axis for restraining. The mandible was then positioned for near occlusion at the incisors to best simulate biting a smaller-sized object.

We exported the cranial meshes and converted to them to FEMs (volume meshes) using 3-Matic (Materialise v.18.0). Each model consisted of approximately 1–2 million 3D tetrahedral elements. Models were then imported into Strand7 (v.R3.1.3.a) for finite element modelling. We assigned homogeneous, isotropic material properties of average mammalian bone (Young's modulus: *E*≈20 GPa; Poisson's ratio: ν=0.3) (Figueirido et al., 2014; Mitchell, 2019; Mitchell et al., 2018; Mitchell and Wroe, 2019; Sharp, 2015; Tseng et al., 2011). Homogeneous and isotropic material properties were considered acceptable to assess the relationship between gross cranial morphology and biting performance (Fitton et al., 2015; Strait et al., 2010; Walmsley et al., 2013). As is common practice in comparative FEA studies (Dumont et al., 2005; Mitchell, 2019; Wroe et al., 2007), we emphasise that our results should be considered in a relative context and not as actual *in vivo* strain magnitudes.

We partitioned major jaw adductor muscle groups (masseter, temporalis and pterygoids) using overall proportions for *Potorous* (Warburton, 2009). We sub-divided the proportions of the masseter complex (superficial, intermediate and deep) and pterygoids (medial and lateral) using subunit proportions of a red-necked wallaby (Mitchell et al., 2018). A total muscle force of 100 N was partitioned according to these muscle proportions for all species and a specimen (*A. rufescens* M2750) was randomly chosen (alphanumerically) as the reference specimen to scale muscle forces to cranial volume for all other models. The absolute reference muscle force is arbitrary and does not change the predicted distribution of stress or strain in a structure (Mitchell, 2019; Wroe, 2007; Wroe et al., 2007). The muscle forces were scaled to cranial volume using a 2/3 power rule (Strait et al., 2010) (Table S1).

Muscle plates representing origins and insertions were defined using Geomagic (v.2021). The masticatory muscle forces were applied to cranial plates using BoneLoad (Davis et al., 2010; Grosse et al., 2007). This software orients the forces from the cranial muscle origins to the centroids of their respective insertions, following the curvature of the bone. The loaded plates were imported into Strand7 and zipped to the nodes of their corresponding elements. A single node on the tip of each biting tooth was restrained against translation in the vertical axis: both incisors for a bilateral incisor bite, and a single, right-side P3 premolar or M2 molar for unilateral simulations. A single node at each temporomandibular joint was restrained against translation for all axes. The models were then solved as linear static.

From these initial simulations, we extracted reaction forces from the biting teeth. We then calculated mechanical advantage by dividing the bite reaction force by the input muscle force. To address hypotheses relating to structural biting adaptation, we rescaled the muscle forces to produce equivalent bite forces, relative to size (Fig. 1B,C). To do this, we first calculated a predicted value of equivalent relative bite reaction force for each specimen by multiplying the input muscle forces by an identical value of mechanical advantage, arbitrarily represented by the mean mechanical advantage of all models obtained from the initial simulations (Table S2, Fig. S1). Dividing this predicted bite reaction force by the initial bite reaction force of the first simulations gives the scaling factor to multiply all initial muscle forces by. The scaling factor is therefore given by the following equation:
(1)

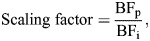
where BF_p_ is the predicted bite reaction force for a given model calculated from the mean mechanical advantage of all models and BF_i_ is the bite reaction force of the initial simulation of the same model. We then multiplied all initial muscle forces by the scaling factor. This method is similar in aim to rescaling the models to the same size and applying muscle forces that result in the same bite reaction force for a given size (e.g. Attard et al., 2011; Oldfield et al., 2011; Wroe et al., 2010); however, we chose this approach because it allows for the same models to be used in all our scaling scenarios. This procedure corrected for the effects mechanical advantage on strain magnitudes during equivalent bites. Differences in mean strain under these conditions should therefore only reflect differences in how well a given cranial morphology deforms, or bends, during a given bite reaction force, relative to size (Dumont et al., 2009; Mitchell et al., 2018; Smith et al., 2015a).

For hypotheses concerning the comparative ability of different animals to withstand identical bites in the wild, we needed the models to produce equivalent absolute bite forces. In this case, predicted bite reaction forces were standardised to the mean value of all bite reaction forces from the initial simulations (Fig. 1D). The precise value used for equivalent absolute bite force is arbitrary in a comparative context, but using the mean bite reaction force allowed us to use the same colouration scales in all visualisations. The scaling factor for this method is given by the same equation as above, but with the predicted bite reaction force being the mean bite reaction force from the initial simulations (Table S3, Fig. S1). We emphasise that the only changes made to the models across these three sets of simulations are the magnitudes of muscle forces applied. All muscle forces and rescaled muscle forces applied to all models are available in the Tables S1–S3.

We used von Mises strain, described in microstrain (µε), to represent deformation and visualised this for specific cases using von Mises heatmaps, set to a threshold of 15 µε. However, the comparative results obtained can also hold true for stress in homogeneous, isotropic models, because stress and strain are directly proportional under such conditions. We extracted strain magnitudes from all elements of each model and created custom R code (http://www.R-project.org/[Bibr JEB249747C40]) to automatically remove the upper 1% of strain values, because these typically relate to artifactually inflated regions close to the restraints (Marcé-Nogué et al., 2017; Walmsley et al., 2013). We plotted histograms of each simulation that depicted mean von Mises strain for each model. A higher degree of mean strain indicates greater deformation of the model during the simulated activity.

## RESULTS AND DISCUSSION

**Fig. 2.**
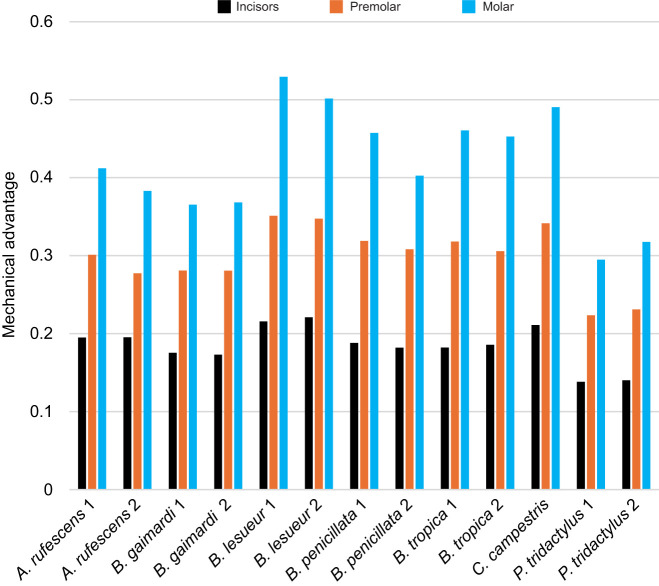
**Mechanical advantage across all simulated bite scenarios.** Data are shown for a bilateral incisor bite (I1) and a bilateral, right-side P3 premolar and unilateral, right side M2 molar for each of the seven study species (13 individuals).

Potoroos and bettongs had a high degree of variation in mechanical advantage (Fig. 2). Across all simulations, the highest mechanical advantage was seen in *B. lesueur*, closely followed by *C. campestris*. The lowest mechanical advantage was seen in *P. tridactylus*. This variation in mechanical advantage means that under the initial muscle loading scenarios, all species were producing bites of different magnitude, relative to their size.

**Fig. 3.**
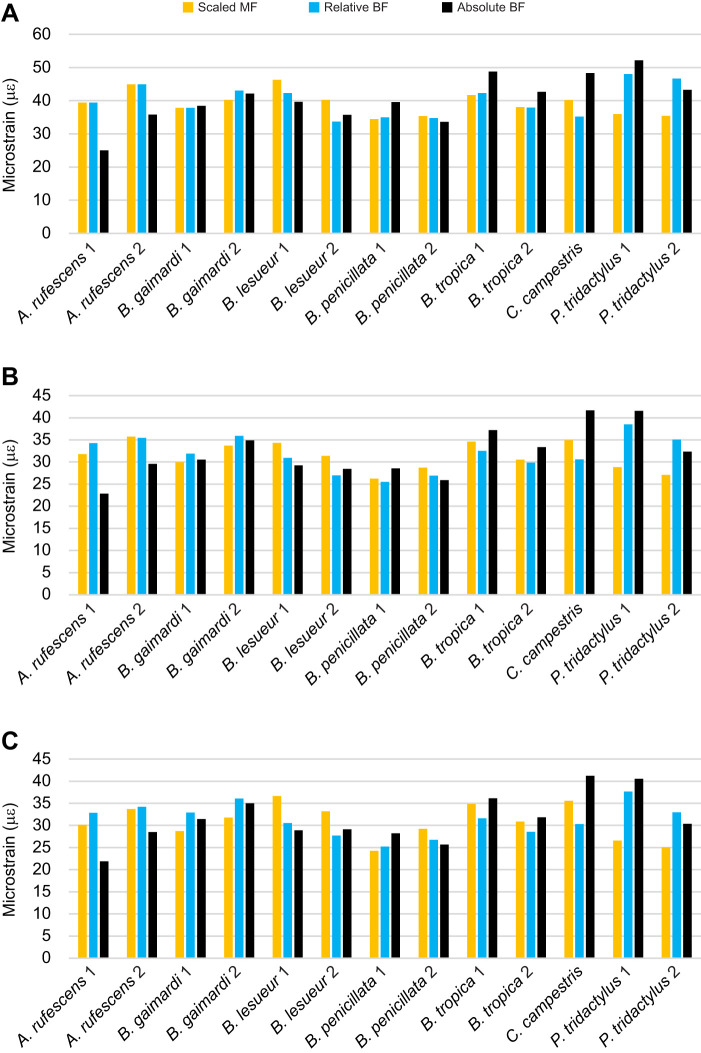
**Mean element microstrain of all simulations.** (A) Incisor bite, (B) P3 premolar bite and (C) M2 molar bite simulation. MF, muscle force; BF, bite force.

As we predicted, lower mechanical advantage in *P. tridactylus* resulted in lower deformation under initial loading conditions with standardised muscle force. Rescaling the muscle forces to produce equivalent relative bite forces (standardised mechanical advantage) both decreased the strain in models with high mechanical advantage and increased the strain of models with low mechanical advantage. This was particularly obvious in *P. tridactylus* (Fig. 3), which needed up to 42% more input muscle force to match the predicted relative bite reaction forces of the sample (Table S2).

**Fig. 4.**
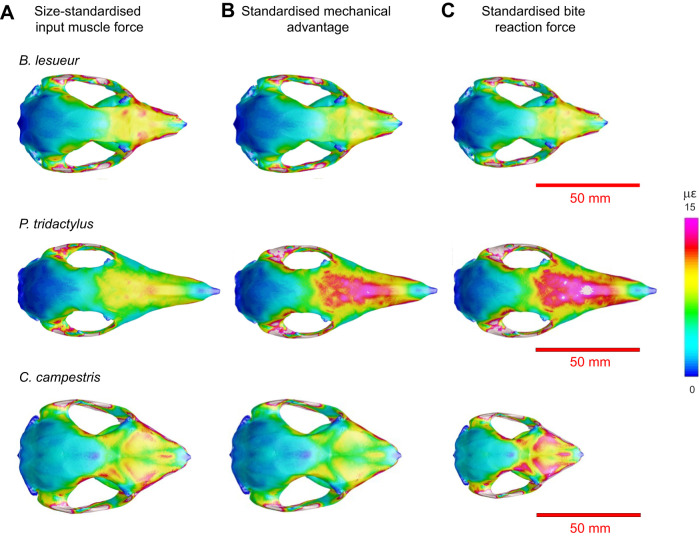
**Examples of von Mises microstrain maps showing the influence that variation in mechanical advantage and size can have on strain or stress magnitude.** Input muscle forces applied represent (A) standardised relative input muscle force through scaling to cranial size (size independent), (B) standardised mechanical advantage by rescaling initial muscle forces from A to produce equivalent relative bite reaction forces (size independent) and (C) standardised absolute bite reaction forces (size dependent) for three species (from top to bottom: *Bettongia lesueur*, *Potorous tridactylus* and *Caloprymnus campestris*).

In Fig. 4, we present dorsal views of von Mises strain heatmaps of some examples that highlight contrasting results in comparative strain magnitudes. Both of the long-faced *P. tridactylus* models had about 4% lower mechanical advantage than the sample average during incisor biting (Table S2), and both models experienced greater mean strain (33.57% and 33.34% greater, respectively) during simulations with standardised mechanical advantage than during the initial simulations of standardised input muscle force (see Fig. 3). Through these notable deviations, the changes in applied forces reversed the comparative patterns of mean strains (Fig. 4), such that the most deformation (indicated by mean strain) was then observed in *P. tridactylus*, while the least was seen in *C. campestris*, *B. penicillata* and one of the *B. lesueur* specimens. The overall interspecific patterns across incisor, premolar and molar bite simulations were mostly consistent despite different magnitudes; however, *C. campestris* performed better during incisor biting. Among *Bettongia*, higher mean strains were consistently found in *B. gaimardi*, *B. tropica* and one of the *B. lesueur* specimens.

Rescaling the muscle forces to produce equivalent absolute bite forces gave similar results for most species, with the high mean strain still found in *P. tridactylus*. But there was a noticeable shift in the performance of the much smaller *C. campestris*, which this time experienced similar mean strain to *P. tridactylus* under this loading regime (Figs 3 and 4). *Caloprymnus campestris* had a cranial volume that was 77% of the next smallest cranium in the sample (*B. tropica*), and 34% of the largest (*A. rufescens*) (Table S1). As an example to demonstrate the impact of its small size, when muscles were rescaled to produce equivalent absolute bite forces at the incisors, this increased the mean strain of *C. campestris* by 20.19% from the initial simulation of standardised input muscle force. Taken together, the two methods of rescaling muscle force offered strikingly contrasting results for *C. campestris* incisor bite simulations, with standardised absolute bite force producing 46.33% more mean strain than standardised relative bite force (Fig. 3). Fig. 3 also identifies some noticeable intraspecific variation in mean strain relating to size variation of the specimens, particularly in *A. rufescens* and *P. tridactylus*; however, these do not appreciably impact overall interspecific comparative patterns – at least in this sample.

### Summary and conclusions

**Fig. 5.**
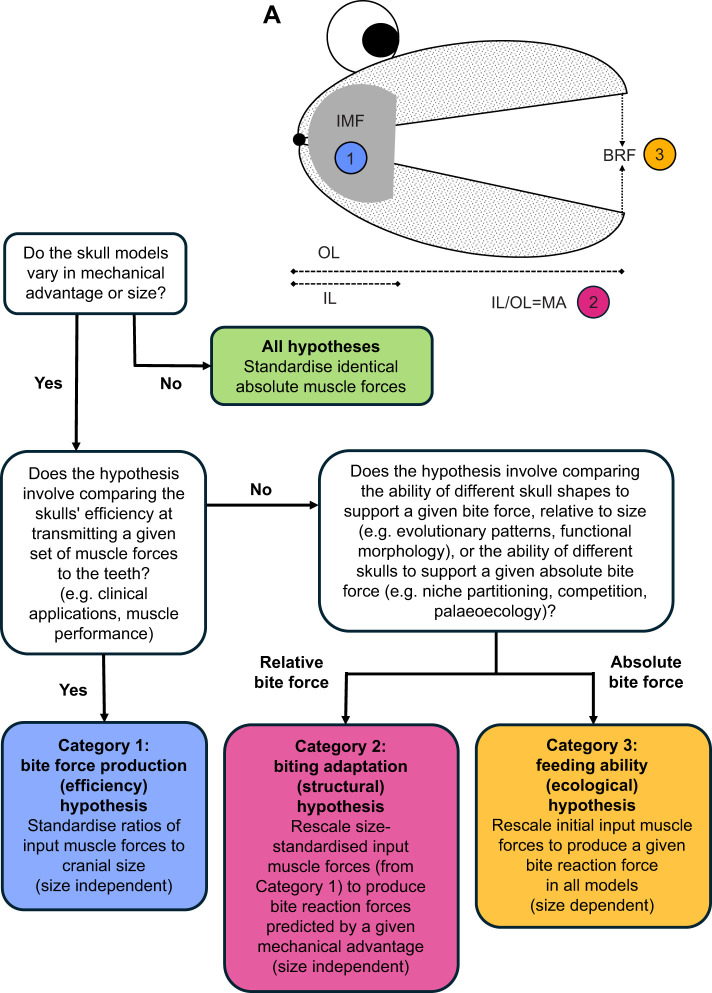
**Proposed methods of muscle scaling for each hypothesis category of feeding biomechanics.** (A) The three points of standardisation on a stylised skull: (1) input muscle force (IMF); (2) mechanical advantage (MA; in-lever IL/out-lever OL); and bite reaction force (BRF). (B) A framework for deciding the appropriate method of muscle scaling to produce reliable stress or strain magnitudes for different hypotheses. Notably, muscle forces for Category 2 must be rescaled from muscle forces of Category 1 in order to maintain underlying ratios of muscle forces and skull size (i.e. size independence) in the correction.

In this study, we focused on methods of jaw muscle scaling in comparative FEA of skulls. We highlighted three standardisations in biting comparisons that each require unique approaches to muscle scaling: input muscle force, mechanical advantage and bite reaction force. Using a sample of potoroid marsupial skulls with appreciable variation in mechanical advantage and size, we show here that each method produces unique results of comparative mean strain, but all are informative for specific cases. Based on our results, we propose that studies aimed at answering questions in the area of feeding biomechanics can be partitioned into three major categories, which each require a specific method of muscle scaling: those interested in comparing: (1) bite force production (system efficiency), through standardising input muscle force to cranial size; (2) biting adaptation (skull structure), via standardising mechanical advantage in muscle forces previously scaled to skull size; or (3) feeding ability (ecology) by standardising bite reaction forces. This framework is presented in Fig. 5.

The first consideration in this framework is whether the models vary in mechanical advantage or size. This is an important first step because there are comparative FEA studies that involve alterations to a single specimen that do not necessarily affect that specimen's mechanical advantage or size (e.g. Tanner et al., 2008). For many taxa studied, as is the case for several species in this study, differences in mechanical advantage or size are not so great as to change comparative results obtained between the methods; therefore, standardising input muscle force alone would be acceptable. However, if there are appreciable differences in mechanical advantage or size between the specimens being modelled, we show that results for the three methods can deviate from each other. We therefore suggest that each of these three major categories might be best examined with their own scaling method. Standardising one of these three conditions (input muscle force, mechanical advantage or bite reaction force) produces variation in the other two conditions, potentially leading to differential results of comparative stress or strain. We have shown that, in some cases, these differences can be substantial enough to produce opposite comparative patterns.

Importantly, methods 2 and 3 require an initial set of simulations for calculating mechanical advantage and rescaling of muscle forces to produce equivalent relative or absolute bite reaction forces. Standardising of mechanical efficiency (Category 2 method) requires the initial simulations to first be scaled to cranial size (Category 1 method). This is to maintain size-independent muscle scaling across specimens, upon which variation in bite reaction force due to variation in mechanical advantage is removed. Without this crucial step, bite reaction forces predicted from standardised mechanical advantage will not represent size-independent assessments of shape performance. However, the initial input muscle forces used for standardising of bite reaction forces (Category 3 method) are arbitrary for subsequent rescaling, because the predicted bite reaction forces are constant and not influenced by size variation.

The initial simulations, with size-standardised input muscle forces applied, provide an estimation of how efficiently different skulls convert a given amount of muscle force to bite force and disperse stress and strain. This method was useful in this study to obtain the mechanical advantage values for rescaling in the two subsequent methods. But our simulations also showed that lower strain magnitudes were found in species with low mechanical advantage. With the understanding that lower strain is an indication of improved bite force capacity (Dumont et al., 2011a; Mitchell, 2019; Wroe, 2010), use of this scaling method might have led to the conclusion that longer-faced species – such as the long-nosed potoroo in our sample – are better adapted for forceful biting.

However, when rescaling muscle forces to produce equivalent relative bite forces, through standardising mechanical advantage, the patterns of the initial simulations were reversed, with the least efficient skulls exhibiting higher mean strain, as would be expected for the longer faced species that feed on high proportions of soft fungi. With muscle forces scaled to represent standardised mechanical advantage, the species with higher mechanical advantage tended to experience lower strain and those with lower mechanical advantage tended to produce greater mean strain magnitudes, when compared with their initial simulations. This reflects the need for skulls with lower mechanical advantage to provide more input muscle force to produce equivalent bites, relative to size (Mitchell et al., 2018, 2024c). Accordingly, greater stress and strain will be dispersed throughout the facial skeleton. In contrast, skulls with greater mechanical advantage need less muscle force to produce equivalent bites, so mean strain will be reduced. This means that the initial scaling method alone is likely not the most appropriate approach to compare stress or strain magnitudes relating to biting adaptation, owing to the confounding effects of mechanical advantage on bite reaction forces. Hypotheses based on comparing how well skulls disperse a given input force to the joints, teeth and throughout the overall structure do, however, still exist. For example, more clinically oriented questions relating to stress or deformation experienced by the facial skeleton under specific loading (e.g. for dentistry or facial prosthetics) can be best answered with the initial muscle scaling method. These kinds of questions fall under the bite force production category (Category 1). However, interspecific studies using comparative FEA of skulls tend to be more interested in how skull shape influences biting performance (Category 2). We showed that these questions are better assessed by rescaling muscle forces to produce equivalent relative bite forces through standardising of mechanical advantage.

Simulations with standardised input muscle force revealed that the extinct *C. campestris* had a highly efficient skull compared with other potoroids, well-shaped to effectively transfer muscle force to bite force. Simulations of standardised mechanical advantage further indicated that *C. campestris* had a skull shape well suited to harder biting, with strain magnitudes similar to those of other efficient skulls of *Bettongia* spp. Operating under the assumption that shape alone is indicative of functional adaptation, we might have concluded that *C. campestris* was adapted for more forceful biting and consider the results an indication of a diet high in mechanically resistant foods. However, to compare the ability of the skulls to accommodate a specific bite in an ecological context (Category 3 hypothesis), we then rescaled muscle forces to produce equivalent absolute bite reaction forces, simulating each individual biting a similar food item. Under these conditions, *C. campestris* performed poorly relative to other species with similarly efficient skulls, with mean strain more comparable to the mechanically inefficient skulls of *P. tridactylus*. This suggests that the compact appearance and high mechanical advantage of the skull of *C. campestris* are not adaptations for more forceful biting in an absolute sense, but instead compensated for its considerably smaller size. This would have allowed it to feed on more mechanically similar foods to larger species (Mitchell et al., 2024b), but not to the extent of specialising on particularly resistant foods.

*Caloprymnus campestris* is considered to have been largely herbivorous (mainly phytophagous) (Finlayson, 1932), but remains of beetles have also been found in gut contents (Flannery and Kendall, 1990). Our results thus support a phytophagous diet previously identified for this species, but further predict that *C. campestris* likely had a diet dominated by softer, higher-quality plant material, such as forbs and fresh leaf material. Supplementing the diet with insects is also probable given this is common among potoroids. The higher performance of incisor biting and lower performance of premolar biting in this species suggests a greater importance of incisal cropping in this species, rather than premolar slicing as is commonly employed for most food processing in other potoroid species and browsing marsupials (Sanson, 1989). However, this might also be a compensatory effect – a smaller skull would require a wider gape angle to bite onto a food item of a given thickness, which could render incisor biting the only option for the initial breakdown of larger items. These considerations would likely have been missed if solely using the other two approaches to scaling muscle forces.

Scaling muscle forces to produce equivalent absolute bite forces also carries some additional considerations. For instance, size influences strain magnitude within species as well as across species. During bites of equivalent absolute magnitude, the larger specimens of both *A. rufescens* and *P. tridactylus* experienced lower strain. Intraspecific variation in size should therefore be considered when assessing model performance for simulations of absolute bite force production. This might also open up the methodology to new considerations of how ontogeny, sexual dimorphism and size variation in general impact aspects of feeding ecology, such as niche differentiation and competition.

## Supplementary Material

10.1242/jexbio.249747_sup1 Supplementary information
