# Supplementary material for: Testing hypotheses of skull function with comparative finite element analysis: three methods reveal contrasting results
Source: J Exp Biol. 2025 Feb 25;228(4):JEB249747. doi: 10.1242/jeb.249747 (PMC11928056; doi:10.1242/jeb.249747)
Supplement: Supplementary information [file jexbio-228-249747-s1.pdf]

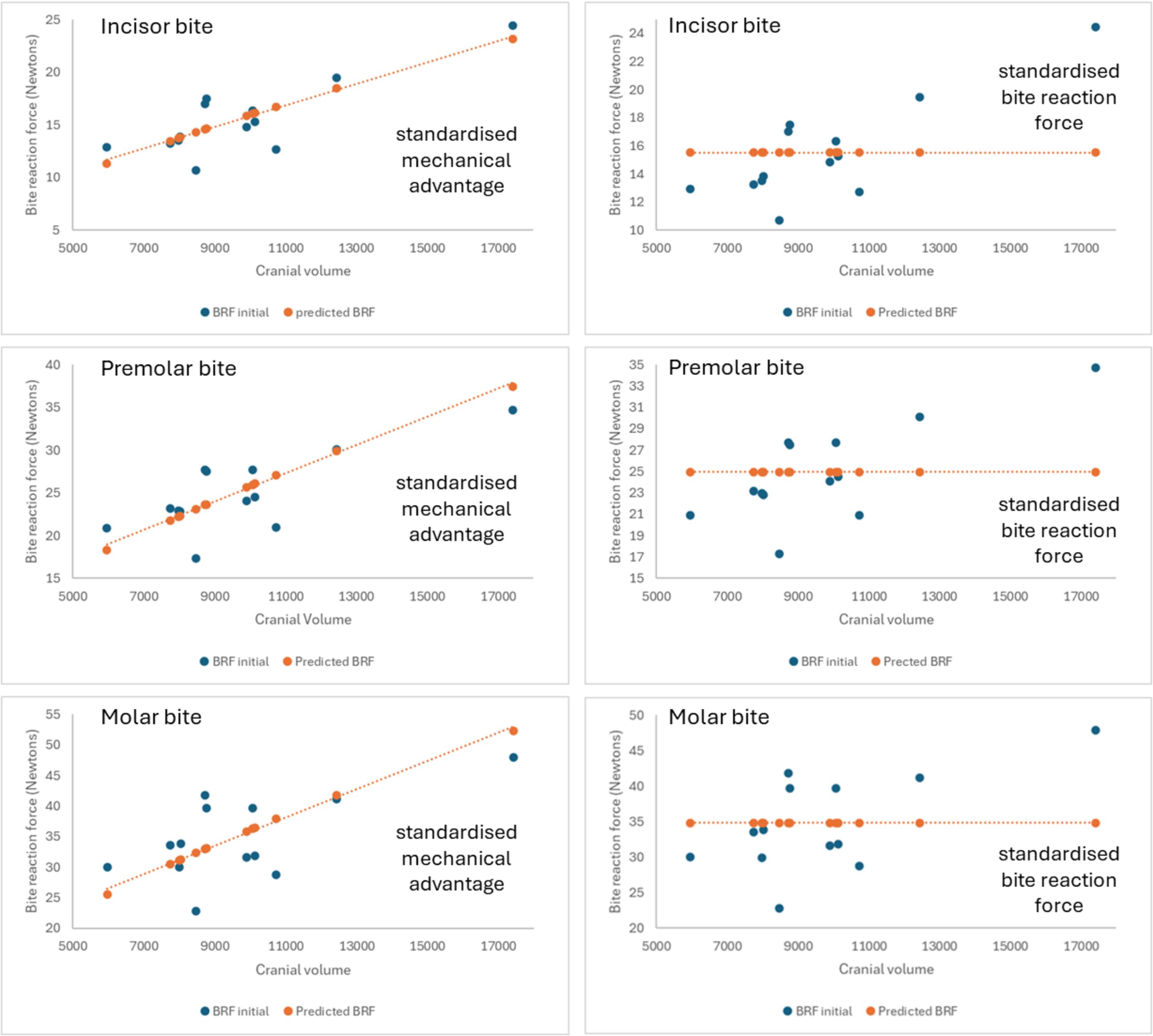

Fig. S1. Visualisations of muscle rescaling.

Table S1. Initial muscle scaling to standardise input muscle force using a 2/3rd power rule of cranial volume (forces for each muscle in Newtons). massd = deep masseter, massi = intermediate masseter, masss = superficial masseter, pterl = lateral pterygoid, pterm = medial pterygoid, temp = temporalis, zyg = zygomaticomandibularis. Listed forces were applied to both sides of the cranium.

Specimen	Volume	Scale	massd	massi	masss	pterl	pterm	temp	zyg	total
Aruf_M2750	12447.79	1	5.45	8.54	7.94	0.94	7.56	13.75	5.82	100
Aruf_M12026	17425.74	1.251409	6.820179	10.68703	9.936188	1.176325	9.460652	17.20687	7.283201	125.1409
Bgai_M7384	10136.22	0.872012	4.752466	7.446983	6.923776	0.819691	6.592411	11.99017	5.07511	87.20121
Bgai_M7389	9897.99	0.858295	4.677707	7.329838	6.814861	0.806797	6.488709	11.80155	4.995276	85.82948
Bles_M1705	8726.37	0.789152	4.300881	6.739362	6.26587	0.741803	5.965992	10.85085	4.592867	78.91524
Bles_M18492	8772.56	0.791935	4.316044	6.763122	6.287962	0.744419	5.987026	10.8891	4.60906	79.19347
Bpen_M8285	10069.09	0.868158	4.731459	7.414067	6.893172	0.816068	6.563272	11.93717	5.052678	86.81577
Bpen_11252	7991.36	0.744193	4.055852	6.355409	5.908893	0.699541	5.626099	10.23265	4.331204	74.4193
Btro_JM10030	7747.19	0.728956	3.97281	6.225284	5.78791	0.685219	5.510907	10.02314	4.242524	72.89559
Btro_JM12495	8033.064	0.74678	4.069951	6.3775	5.929432	0.701973	5.645656	10.26822	4.346259	74.67799
Ccam_M3257	5964.31	0.61232	3.337145	5.229215	4.861823	0.575581	4.629141	8.419403	3.563704	61.23202
Ptri_M7381	8472.04	0.773744	4.216903	6.607771	6.143525	0.727319	5.849503	10.63898	4.503188	77.37437
Ptri_M9013	10737.57	0.906169	4.93862	7.738682	7.194981	0.851799	6.850637	12.45982	5.273903	90.61689

Table S2. Rescaling input muscle forces with standardised mechanical advantage. IMF = initial muscle force, BRF = bite reaction force, MA = mechanical advantage, RMF = rescaled estimated muscle force. All muscle forces in table S1 were multiplied by the scaling factor for each species to achieve the total RMF.

				Incisor Bite				
Specimen	Vol	logVol	IMF	Initial BRF	MA	Predicted BRF	Scaling factor	RMF
Aruf_M2750	12447.79	4.10	100.00	19.49	0.19	18.48	0.95	94.82
Aruf_M12026	17425.74	4.24	125.14	24.45	0.20	23.13	0.95	118.37
Bgai_M7384	10136.22	4.01	87.20	15.30	0.18	16.12	1.05	91.85
Bgai_M7389	9897.99	4.00	85.83	14.84	0.17	15.86	1.07	91.74
Bles_M1705	8726.37	3.94	78.92	17.02	0.22	14.58	0.86	67.62
Bles_M18492	8772.56	3.94	79.19	17.50	0.22	14.64	0.84	66.23
Bpen_M8285	10069.09	4.00	86.82	16.33	0.19	16.04	0.98	85.30
Bpen_11252	7991.36	3.90	74.42	13.54	0.18	13.75	1.02	75.59
Btro_JM10030	7747.19	3.89	72.90	13.27	0.18	13.47	1.02	74.00
Btro_JM12495	8033.06	3.90	74.68	13.86	0.19	13.80	1.00	74.36
Ccam_M3257	5964.31	3.78	61.23	12.92	0.21	11.32	0.88	53.63
Ptri_M7381	8472.04	3.93	77.37	10.70	0.14	14.30	1.34	103.40
Ptri_M9013	10737.57	4.03	90.62	12.71	0.14	16.75	1.32	119.40
**Mean**					0.18			
				**Premolar bite**				
Aruf_M2750	12447.79	4.10	100.00	30.11	0.30	29.89	0.99	99.26
Aruf_M12026	17425.74	4.24	125.14	34.70	0.28	37.40	1.08	134.89
Bgai_M7384	10136.22	4.01	87.20	24.50	0.28	26.06	1.06	92.76
Bgai_M7389	9897.99	4.00	85.83	24.10	0.28	25.65	1.06	91.36
Bles_M1705	8726.37	3.94	78.92	27.70	0.35	23.59	0.85	67.20
Bles_M18492	8772.56	3.94	79.19	27.51	0.35	23.67	0.86	68.14
Bpen_M8285	10069.09	4.00	86.82	27.68	0.32	25.95	0.94	81.38
Bpen_11252	7991.36	3.90	74.42	22.93	0.31	22.24	0.97	72.19
Btro_JM10030	7747.19	3.89	72.90	23.19	0.32	21.79	0.94	68.49
Btro_JM12495	8033.06	3.90	74.68	22.84	0.31	22.32	0.98	72.98
Ccam_M3257	5964.31	3.78	61.23	20.91	0.34	18.30	0.88	53.59
Ptri_M7381	8472.04	3.93	77.37	17.30	0.22	23.13	1.34	103.43
Ptri_M9013	10737.57	4.03	90.62	20.93	0.23	27.08	1.29	117.26
**Mean**					0.30			
				**Molar bite**				
Aruf_M2750	12447.79	4.10	100.00	41.20	0.41	41.81	1.01	101.48
Aruf_M12026	17425.74	4.24	125.14	47.93	0.38	52.32	1.09	136.61
Bgai_M7384	10136.22	4.01	87.20	31.85	0.37	36.46	1.14	99.81
Bgai_M7389	9897.99	4.00	85.83	31.60	0.37	35.88	1.14	97.46
Bles_M1705	8726.37	3.94	78.92	41.78	0.53	32.99	0.79	62.32
Bles_M18492	8772.56	3.94	79.19	39.72	0.50	33.11	0.83	66.02
Bpen_M8285	10069.09	4.00	86.82	39.70	0.46	36.30	0.91	79.38
Bpen_11252	7991.36	3.90	74.42	29.95	0.40	31.11	1.04	77.31
Btro_JM10030	7747.19	3.89	72.90	33.57	0.46	30.48	0.91	66.17
Btro_JM12495	8033.06	3.90	74.68	33.81	0.45	31.22	0.92	68.97
Ccam_M3257	5964.31	3.78	61.23	30.03	0.49	25.60	0.85	52.20
Ptri_M7381	8472.04	3.93	77.37	22.81	0.29	32.35	1.42	109.72
Ptri_M9013	10737.57	4.03	90.62	28.78	0.32	37.89	1.32	119.29
**Mean**					0.42			

Table S3. Rescaling input muscle forces with standardised bite reaction force. IMF = initial muscle force, BRF = bite reaction force, MA = mechanical advantage, RMF = rescaled estimated muscle force. All muscle forces in table S1 were multiplied by the scaling factor for each species to achieve the total RMF.

Incisor bite								
**Specimen**	**Vol**	**logVol**	**IMF**	**Initial BRF**	**MA**	**Predicted BRF**	**Scaling factor**	**RMF**
Aruf_M2750	12447.79	4.10	100.00	19.49	0.19	15.53	0.80	79.68
Aruf_M12026	17425.74	4.24	125.14	24.45	0.20	15.53	0.64	79.49
Bgai_M7384	10136.22	4.01	87.20	15.30	0.18	15.53	1.02	88.51
Bgai_M7389	9897.99	4.00	85.83	14.84	0.17	15.53	1.05	89.82
Bles_M1705	8726.37	3.94	78.92	17.02	0.22	15.53	0.91	72.01
Bles_M18492	8772.56	3.94	79.19	17.50	0.22	15.53	0.89	70.28
Bpen_M8285	10069.09	4.00	86.82	16.33	0.19	15.53	0.95	82.56
Bpen_11252	7991.36	3.90	74.42	13.54	0.18	15.53	1.15	85.36
Btro_JM10030	7747.19	3.89	72.90	13.27	0.18	15.53	1.17	85.31
Btro_JM12495	8033.06	3.90	74.68	13.86	0.19	15.53	1.12	83.68
Ccam_M3257	5964.31	3.78	61.23	12.92	0.21	15.53	1.20	73.60
Ptri_M7381	8472.04	3.93	77.37	10.70	0.14	15.53	1.45	112.30
Ptri_M9013	10737.57	4.03	90.62	12.71	0.14	15.53	1.22	110.72
**Mean**				15.53				
**Premolar bite**								
Aruf_M2750	12447.79	4.10	100.00	30.11	0.30	24.95	0.83	82.86
Aruf_M12026	17425.74	4.24	125.14	34.70	0.28	24.95	0.72	89.98
Bgai_M7384	10136.22	4.01	87.20	24.50	0.28	24.95	1.02	88.80
Bgai_M7389	9897.99	4.00	85.83	24.10	0.28	24.95	1.04	88.86
Bles_M1705	8726.37	3.94	78.92	27.70	0.35	24.95	0.90	71.08
Bles_M18492	8772.56	3.94	79.19	27.51	0.35	24.95	0.91	71.82
Bpen_M8285	10069.09	4.00	86.82	27.68	0.32	24.95	0.90	78.25
Bpen_11252	7991.36	3.90	74.42	22.93	0.31	24.95	1.09	80.98
Btro_JM10030	7747.19	3.89	72.90	23.19	0.32	24.95	1.08	78.43
Btro_JM12495	8033.06	3.90	74.68	22.84	0.31	24.95	1.09	81.58
Ccam_M3257	5964.31	3.78	61.23	20.91	0.34	24.95	1.19	73.06
Ptri_M7381	8472.04	3.93	77.37	17.30	0.22	24.95	1.44	111.59
Ptri_M9013	10737.57	4.03	90.62	20.93	0.23	24.95	1.19	108.02
**Mean**				24.95				
**Molar bite**								
Aruf_M2750	12447.79	4.10	100.00	41.20	0.41	34.83	0.85	84.54
Aruf_M12026	17425.74	4.24	125.14	47.93	0.38	34.83	0.73	90.94
Bgai_M7384	10136.22	4.01	87.20	31.85	0.37	34.83	1.09	95.35
Bgai_M7389	9897.99	4.00	85.83	31.60	0.37	34.83	1.10	94.59
Bles_M1705	8726.37	3.94	78.92	41.78	0.53	34.83	0.83	65.79
Bles_M18492	8772.56	3.94	79.19	39.72	0.50	34.83	0.88	69.45
Bpen_M8285	10069.09	4.00	86.82	39.70	0.46	34.83	0.88	76.17
Bpen_11252	7991.36	3.90	74.42	29.95	0.40	34.83	1.16	86.55
Btro_JM10030	7747.19	3.89	72.90	33.57	0.46	34.83	1.04	75.62
Btro_JM12495	8033.06	3.90	74.68	33.81	0.45	34.83	1.03	76.94
Ccam_M3257	5964.31	3.78	61.23	30.03	0.49	34.83	1.16	71.02
Ptri_M7381	8472.04	3.93	77.37	22.81	0.29	34.83	1.53	118.13
Ptri_M9013	10737.57	4.03	90.62	28.78	0.32	34.83	1.21	109.67
**Mean**				34.83				
